# Is multimorbidity associated with higher risk of falls among older adults in India?

**DOI:** 10.1186/s12877-022-03158-5

**Published:** 2022-06-04

**Authors:** Manish Barik, Sushree Nibedita Panda, Sweta Sulagna Tripathy, Abhinav Sinha, Shishirendu Ghosal, Ardhendhu Sekhar Acharya, Srikanta Kanungo, Sanghamitra Pati

**Affiliations:** ICMR-Regional Medical Research Centre, Bhubaneswar, Odisha India

**Keywords:** Fall, Multimorbidity, LASI, India, Older adults, Ageing

## Abstract

**Background:**

Fall, a multifaceted health condition, is one of the major causes of mortality among older adults. Rapid ageing and increased multimorbidity in low-and middle-income countries (LMICs), including India, might elevate the risk of fall. Although, fall is associated with significant healthcare utilization, it still remains an under-recognized public health issue. This accentuates a need for evidence on fall to integrate it into existing healthcare programs, a gap in geriatric care. The present study aimed to assess the association of fall with multimorbidity among older adults in India.

**Methods:**

We included 28,567 participants aged ≥ 60 years from Longitudinal Ageing Study in India (LASI), wave-1 conducted during 2017–19. Descriptive statistics were used to compute the prevalence of self-reported falls along with 95% confidence interval as a measure of uncertainty. The association between falls and multimorbidity was assessed by multivariable logistic regression and presented as an adjusted odds ratio (AOR).

**Results:**

The prevalence of falls was 12.5%, being higher among women (13.6% vs. 11.4%) than men. The major determinants of fall were females, rural residents and smokeless tobacco use. We observed multimorbidity [AOR: 1.29 (1.14–1.46)] to be significantly associated with falls.

**Conclusion:**

Falls are commonly prevalent among older adults having multimorbidity as its important predictor. Existing health programs should incorporate falls as an important part of geriatric care. Additionally, primary health care facilities should be strengthened to provide comprehensive care for injuries sustained due to falls.

**Supplementary Information:**

The online version contains supplementary material available at 10.1186/s12877-022-03158-5.

## Background

Owing to the demographic transitions and advancements in healthcare, there is a significant rise in the ageing population among low-and middle-income countries (LMICs). Projections indicate the population of older adults aged ≥ 60 years may increase to twice with more than 80% of the population living in LMICs by 2050 [1]. Among LMICs, India is the second most populous country with over a billion people according to the population census of India, 2011 [2]. There are nearly 103 million adults aged ≥ 60 years in India and the projections estimate that this group will further rise to 19.5% of the population or 319 million by 2050 [3]. Fall is one of the main causes of injuries and an under-recognized public health issue among older adults living in LMICs such as India.

Around one-third of adults aged ≥ 65 years have experienced at least one fall every year [4]. Falls are also a second major cause of death in adults aged 60 years and above after road traffic accident injuries [5]. Falls lead to 10% to 15% of all critical care facility visits and are the underlying cause of 20% to 30% of mild-to-severe injuries with more than 50% of injury related hospitalizations among individuals aged 65 years and above [6]. The major clinical conditions for fall-associated hospitalizations are hip fractures, brain trauma, and upper extremity traumas. Evidence suggests, the period of hospitalization after fall-related injuries ranges from four to fifteen days and may be extended when associated with frailty, hip fractures, and advancing age. 30% to 55% of adults aged 60 years and above fear a fall, and above 30% report limiting their daily activities [7]. Injuries due to falls are considered as unpreventable and significantly higher in number. Globally, 38 million disability-adjusted life years (DALY) are lost due to falls [5]. Out of eighty percent fall related fatalities, 60% deaths were reported in Western Pacific and South East Asia [5].

Multimorbidity described as the presence of two or more chronic illnesses in an individual without defining the index disease [8], has become common in India [9]. Multimorbidity often necessitates increased healthcare utilization which may further rise among patients with fall [10]. Previous studies from high income countries (HICs) suggest multimorbid individuals aged 60 years and above were at a greater risk of falls than others [11, 12]. The age related chronic conditions often leads to sensory deficits and mobility issues which may increase the chances of incurring a fall [11]. Gait, use of walking aids, vertigo, conditions like Parkinson’s disease, polypharmacy associated with multimorbidity can be the major risk factors of falls among older adults. But, there is a paucity of literature on association between multimorbidity and fall in LMICs with no such study in India. Notably, even though fall is prominent among older adults, still it is not included within existing health programmes such as National Program for Health Care of Elderly.

Therefore, generating evidence on falls can assist in strengthening geriatric care and further help in enhancing the quality of life among this group. Additionally, it could guide existing healthcare programs and future policy for ageing population. Therefore, we aimed to assess the association between fall and multimorbidity among adults aged ≥ 60 years in India using data from Longitudinal Ageing Study (LASI), wave1.

## Method

### Overview of data

This study was carried out using data from the first wave of LASI, conducted in 2017–18. LASI is a national survey to scientifically investigate the health, economics, social determinants and repercussion of population ageing in India. The first wave covered a baseline sample of 72,250 participants aged ≥ 45 years along with their partners (irrespective of age) from all states and union territories of India except Sikkim.

To reach the final unit of observation, LASI used a multi-stage stratified cluster sampling technique incorporating three and four stages of sampling for rural and urban areas respectively. LASI report available on its website provides extensive details of sample design, survey instruments, data collection strategies, response rates and fieldwork [13].

### Study participants and sample size

In LASI, a total of 72,250 participants were surveyed. We included 28,567 participants aged ≥ 60 years whose complete data on individual as well as biomarkers were available after excluding incomplete/missing data (supplementary figure S1).

### Outcome variable

The primary outcome variable of interest fall, was assessed through the self-reported question “Have you fallen down in last two years?” reported in binary as ‘Yes’ or ‘No’. The recurrent falls among the participants was assessed through the question “number of times fallen down in last two years”.

### Explanatory variables

#### Individual characteristics

We used age, sex (male/female), residence, education, caste, region, working status, partner status, and wealth index among individual characteristics. For this study, participants’ age, a continuous variable was categorized as ‘60–69 years’, ‘70–79 years’, and ‘80 years and above’. Residence of the participants were classified as ‘rural’ and ‘urban’. Education was classified as having ‘formal education’ (those who ever attended school) and ‘no formal education’ (those who never attended school). Caste was labelled as ‘scheduled caste (SC)’, ‘scheduled tribe (ST)’, ‘other backward class (OBC)’; general and no caste were clubbed as ‘others’. States were grouped on the basis of their geographic location as ‘North’, ‘Central’, ‘East’, ‘North-East’, ‘West’ and ‘South’ categorized as region. Working status was segregated as ‘currently working’; and those who currently did not work or had never worked in their lifetime were combined under ‘currently not working’. Participants in live-in relationships and currently married were allocated to ‘living with partner’ and those who were separated, divorced, widowed, never married and deserted were grouped as ‘not living without partner’. The economic status of the participants was classified as poorest, poorer, middle, richer and richest based on the monthly per capita expenditure (MPCE).

#### Personal/ behavioral attributes

Alcohol intake among participants was categorized as ‘yes’ and ‘no’. Tobacco consumption was categorized on the basis of the type of tobacco used i.e. ‘smokeless tobacco’, ‘smoking’, both smoke and smokeless tobacco ‘dual use’ and ‘abstainer’.

#### Health attributes

Mean arterial pressure was formulated as 2/3 diastolic pressure + 1/3 systolic pressure. The cutoff for hypertension was fixed as systolic: > 140 mm Hg and diastolic: > 90 mm Hg classified as ‘hypertensive’ and ‘non hypertensive’. Obesity was categorized as ‘non-obese’ and ‘obese’, based on body mass index (BMI) ≥ 25 kg/m^2^ [14].

#### Multimorbidity ascertainment

We used sixteen self-reported chronic illnesses (hypertension, diabetes, cancer, chronic lung disease, chronic heart disease, stroke, bone or joint diseases, neurological or psychiatric problems, hypercholesterolemia, thyroid disease, gastrointestinal problems, chronic renal disease, skin diseases, visual impairment, hearing defect, and obesity to create multimorbidity as a simple count of all conditions in an individual where each condition was scored one.

### Statistical analysis

Data were analyzed using STATA version 16.0 (STATA Corp., Texas) and Microsoft Office Excel for graphs. Descriptive statistics was used to report frequency and proportions of socio-demographic characteristics of the participants and period prevalence of falls. Binary logistic regression was run to assess the association of various participant characteristics with falls. The statistically significant variables (*p* < 0.05) from unadjusted model were included to execute a multivariable logistic regression which determined the association between falls and various correlates, indicated as adjusted odds ratio (AOR) with 95% confidence interval (CI). Further, a separate multivariable logistic regression was run to investigate the association between fall and individual selected chronic conditions. Sampling weights were taken into consideration during analysis for both descriptive and regression models. For all weighted proportions, we added 95% CI as the measure of uncertainty.

### Ethical considerations

Ethical clearance for LASI was obtained from the Indian Council of Medical Research (ICMR), New Delhi, and the International Institute of Population Sciences, Mumbai. LASI obtained individual prior informed written consent from all participants. However, the present study is based on secondary anonymous data acquired from LASI; hence, there is no risk to the participants. The data used is appropriately acknowledged and cited wherever needed.

## Results

### Characteristics of the study population

This study is based on 28,567 participants with an age range from 60 to 116 years and a mean age of 68.7 (± 7.3) years. Nearly two-third of the participants were from rural areas. Most of the participants were 60–69 years of age. Sex distribution of study participants was also almost equal with slight female (51.9%) predilection. Most of the participants lived with their partners. Alcohol was consumed by around 17.3% of the participants (Table 1).

**Table 1:** Unweighted socio-demographic characteristic of study participants

Socio-demographic characteristics	Categories	n (%)
**Age (years)** (*n* = 28,567)	60–69	17,421 (61.0)
	70–79	8262 (28.9)
	≥ 80	2884 (10.1)
**Sex** (*n* = 28,567)	Male	13,743 (48.1)
	Female	14,824 (51.9)
**Residence** (*n* = 28,567)	Rural	19,021 (66.6)
	Urban	9546 (33.4)
**Education** (*n* = 28,567)	Formal Education	13,274 (46.5)
	No formal education	15,293 (53.5)
**Caste** (*n* = 28,340)	Scheduled Caste	4662 (16.5)
	Scheduled Tribe	4732 (16.7)
	Other Backward Class	10,865 (38.3)
	Others	8081 (28.5)
**Working status** (*n* = 28,564)	Currently working	8557 (30.0)
	Currently not working	20,007 (70.0)
**Partner status** (*n* = 28,306)	Living with partner	18,361 (64.9)
	Not living with partner	9945 (35.1)
**Wealth Index** (*n* = 28,567)	Most Deprived	5852 (20.5)
	2	5896 (20.6)
	3	5871 (20.6)
	4	5629 (19.7)
	Most affluent	5319 (18.6)
**Alcohol consumption** (*n* = 28,534)	Yes	4936 (17.3)
	No	23,598 (82.7)
**Tobacco consumption** (*n* = 28,528)	Smoking	4791 (16.8)
	Smokeless	5390 (18.9)
	Dual	1054 (3.7)
	Abstainer	17,293 (60.6)
**Region** (*n* = 28,567)	North	4323 (15.1)
	Central	4860 (17.0)
	East	5325 (18.7)
	North-east	3421 (12.0)
	West	3800 (13.3)
	South	6838 (23.9)

Hypertension (55.8%) was the leading chronic condition among study participants followed by obesity (22.2%) and bone/joint disorders (19.6%). A detailed description of the prevalence of selected chronic conditions is presented in supplementary table S2. The overall prevalence of multimorbidity was around 51.1%.

### Prevalence and distribution of falls

We observed the overall prevalence of falls to be 12.5%. Out of the total, 13.6% of females experienced falls whereas amongst males it was 11.4%. We observed a higher prevalence of falls among adults aged 80 years and above. The detailed description of prevalence of falls across various socio-demographic and behavioural attributes is presented in Table 2.

**Table 2:** Prevalence of falls across various individual attributes

Socio-demographic characteristics	Categories	n, %	(95% CI)
**Age (years)**	60–69	1930, 12.4	(11.9–12.9)
	70–79	929, 12.2	(11.5–13.0)
	≥ 80	371, 14.3	(13.0–15.7)
**Sex**	Male	1438, 11.4	(10.9–12.0)
	Female	1791, 13.6	(13.0–14.2)
**Residence**	Rural	2468, 13.4	(12.9–13.9)
	Urban	762, 10.3	(9.6–11.0)
**Education**	Formal education	1310, 11.7	(11.1–12.3)
	No formal education	1920, 13.2	(12.6–13.8)
**Caste**	Scheduled Caste	647, 13.5	(12.5–14.4)
	Scheduled Tribe	234, 11.1	(9.8–12.5)
	Other Backward Class	1383, 11.8	(11.2–12.4)
	Others	932, 13.4	(12.6–14.3)
**Working status**	Currently working	1103, 13.3	(12.5–14.0)
	Currently not working	2127, 12.2	(11.7–12.7)
**Partner status**	Living with partner	1937, 11.8	(11.3–12.3)
	Not living with partner	1267, 13.9	(13.2–14.6)
**Wealth Index**	Most deprived	646, 11.5	(10.7–12.4)
	2	765, 13.6	(12.7–14.5)
	3	630, 11.8	(10.9–12.7)
	4	647, 12.8	(11.9–13.8)
	Most affluent	542, 13.1	(12.1–14.2)
**Alcohol consumption**	Yes	500, 13.1	(12.0–14.2)
	No	2728, 12.5	(12.1–12.9)
**Tobacco consumption**	Smoking	441, 10.6	(9.7–11.6)
	Smokeless	829, 15.3	(14.4–16.3)
	Dual	105, 12.3	(10.1–14.7)
	Abstainer	1851, 12.1	(11.6–12.6)
**Region**	North	270, 12.8	(11.4–14.3)
	Central	844, 12.2	(11.5–13.0)
	East	897, 15.2	(14.3–16.2)
	North-east	74, 9.8	(7.8–12.2)
	West	652, 15.0	(14.0–16.1)
	South	493, 8.5	(7.8–9.3)

**Fig. 1:**
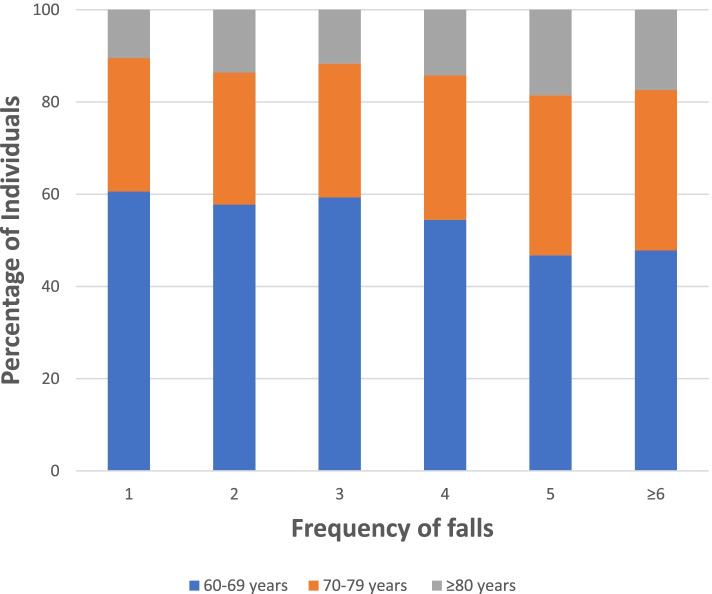
Frequency of falls across various age groups during the last two years

A majority (60.6%) of participants aged 60–69 years experienced at least one fall in the last two years. Amongst respondents aged ≥ 80 years, 18.5% individuals had five falls in last two years. 34.8% of the participants aged 70–79 years reported six or more falls (Fig. 1).

### Association of falls with multimorbidity

Association of falls with various selected chronic conditions determined through a separate multivariable logistic regression (supplementary table S3) showed greater risk [AOR: 1.52 (1.09–2.12)] of falls among participants having hypercholesterolemia. The odds of falls were higher among participants with chronic renal diseases [AOR: 1.50 (1.20–1.87)] followed by gastrointestinal problems [AOR: 1.50 (1.32–1.72)]. Bone and joint diseases [AOR: 1.18 (1.02–1.37)] and stroke [AOR: 1.45 (1.02–2.06)] were found to be significantly associated with falls.

The binary logistic regression identified sex, residence, education, region, caste, living without partner, wealth index, smokeless tobacco and multimorbidity as predictors of fall (Table 3). Age, working status and alcohol consumption were not considered in the multivariable analysis as these were not significant. Multivariable regression model indicated a higher likelihood of falls among females [AOR: 1.16 (1.00–1.34)]. Higher odds of falls was experienced among rural residents [AOR: 1.38 (1.20–1.60)] as compared to urban residents. The risk of falls was highest among participants living in the eastern region as compared to other regions. The odds of experiencing falls was higher among the most affluent group [AOR: 1.21 (1.01–1.46)] as compared to the most deprived group. The likelihood of experiencing falls was highest among smokeless tobacco users [AOR: 1.24 (1.06–1.45)], as compared with smokers and dual tobacco users. Multimorbidity evolved as a strong predictor [AOR: 1.29 (1.14–1.46)] of falls.

**Table 3:** Multivariable regression analysis depicting association of fall with various socio-demographic attributes

Socio-demographic attributes	Categories	Falls		
		**OR (95% CI)**	**AOR (95% CI)**	
**Age (years)**	60–69	Reference		
	70–79	0.98 (0.86–1.11)	Not included in the model	
	≥ 80	1.18 (0.96–1.44)		
**Sex**	Male	Reference		
	Female	1.22 (1.08–1.37)	1.16 (1.00–1.34)	
**Residence**	Rural	1.35 (1.18–1.54)	1.38 (1.20–1.60)	
	Urban	Reference		
**Education**	Formal education	Reference		
	No formal education	1.15 (1.02–1.29)	1.03 (0.90–1.19)	
**Caste**	Scheduled Caste	1.24 (0.99–1.57)	1.25 (0.98–1.59)	
	Scheduled Tribe	Reference		
	Other Backward Class	1.07 (0.87–1.32)	1.20 (0.97–1.49)	
	Others	1.24 (1.00–1.54)	1.25 (0.99–1.57)	
**Working status**	Currently working	1.10 (0.97–1.25)	Not included in the model	
	Currently not working	Reference		
**Partner status**	Living with partner	Reference		
	Living without partner	1.20 (1.06–1.36)	1.17 (1.03–1.33)	
**Wealth Index**	Most Deprived	Reference		
	2	1.21 (1.02–1.44)	1.18 (0.99–1.41)	
	3	1.03 (0.86–1.23)	1.03 (0.86–1.24)	
	4	1.13 (0.94–1.36)	1.14 (0.95–1.38)	
	Most affluent	1.17 (0.98–1.39)	1.21 (1.01–1.46)	
**Alcohol consumption**	Yes	1.06 (0.91–1.24)	Not included in the model	
	No	Reference		
**Tobacco Consumption**	Smoking	0.86 (0.73–1.02)	0.96 (0.80–1.15)	
	Smokeless	1.32 (1.15–1.52)	1.24 (1.06–1.45)	
	Dual	1.02 (0.78–1.33)	1.04 (0.78–1.39)	
	Abstainer	Reference		
**Multimorbidity**	Yes	1.22 (1.08–1.38)	1.29 (1.14–1.46)	
	No	Reference		
**Region**	North	1.58 (1.30–1.92)		1.52 (1.23–1.87)
	Central	1.49 (1.24–1.80)		1.54 (1.28–1.86)
	East	1.93 (1.61–2.30)		1.88 (1.56–2.27)
	North-east	1.17 (0.92–1.48)		1.19 (0.92–1.54)
	West	1.90 (1.55–2.32)		1.94 (1.57–2.40)
	South	Reference		

## Discussion

Fall is a multifactorial and interrelated health condition, most common among older adults. However, there is limited research on falls and their association with multimorbidity. Therefore, we carried out this study to generate comprehensive nationwide evidence on falls among older Indians. The present study assessed the association between fall and multimorbidity among Indians aged ≥ 60 years. We observed the prevalence of falls to be around 12.5% with a slight predilection among females. The various predictors of fall were rural residents, higher economic status and multimorbidity.

The prevalence of falls in this study was 12.5% which is lower than a study reported in community dwelling adults of South Korea (15.9%) [15]. Additionally, it is also lower than a community-based cross-sectional study carried out among respondents aged ≥ 60 years in Maharashtra which reported the prevalence of falls to be around 25% [16]. Nonetheless, the magnitude of fall observed in our study still poses a great challenge for already swamped Indian health system, considering its vast population. Further, falls lead to the development of fear of falling among older adults which may compel them to restrict their activities of daily living. This would also lead to a decline in physical activity, social isolation and depression thus, turning into a vicious circle by further increasing the risk of falls [17].

We observed a female predilection for falls in this study which is in harmony with the outcomes of a prior study conducted among older adults aged 65 years and above in China [18]. The reason behind the differences in the prevalence of falls across gender could be due to a decline in bone mass and weaker quadriceps muscles especially after menopause in females [19, 20]. We found fall to be associated with rural residents which is consistent with the findings of a study based in China which reported a higher incidence of falls among rural residents aged 65 years and over [18]. A significant association between affluent groups with falls was observed in this study which may be due to a sedentary lifestyle with less physical activities among this group. However, our results are in contrast with earlier studies conducted among participants aged ≥ 60 years in India, where the deprived class was found to be associated with falls [21, 22].

We observed the prevalence of multimorbidity to be around 51.1% which is in harmony with the findings of our prior study which estimated the prevalence of multimorbidity to be around 50% among adults aged ≥ 45 years [23, 24]. Interestingly, previous studies are in concurrence with our findings that falls are significantly associated with hearing defects [22, 25]. Additionally, a study conducted among elderly residing in old age homes of Hyderabad found falls to be associated with diabetes, depression and visual impairment which is also consistent with the findings of our study [26]. Furthermore, we observed multimorbidity to be significantly associated with falls which are in line with the outcomes from an Indonesian study conducted among participants aged 60 years and above residing in community and elderly homes [27].

The various underlying mechanisms for falls could be complex and multifactorial such as eye disorders (cataract and glaucoma), depression and the use of anti-depressants, skeletal muscle dysfunction, balance-deficits, restricted activities of daily living, postural oscillations, and gait. Multimorbidity may reduce physical activity, exacerbate disablement and reduce compliance to treatments paving a way for fall. A meta-analysis depicted a strong association between the strength of lower extremity and increased risk of falls [28]. We did not investigate the association of polypharmacy or use of drugs with the risk of falls which needs to be explored further. Here, it is worth noting that to date no study has explored the association between falls and multimorbidity in India which posed a challenge in comparing our outcomes with other related research.

### Implications for policy and practice

Considering India’s rapidly ageing population, it is imperative that the burden of falls among older adults will rise. Additionally, our findings suggest that the high prevalence of multimorbidity will further raise the risk of falling. Therefore, it is pertinent to adopt and implement fall prevention and management programs merging it with the existing geriatric healthcare programs. There is a need to strengthen primary care to provide preventive and curative services for multimorbidity where the newly formed Health and Wellness Centres (HWCs) can act as a window of opportunity. These HWCs should also have fall clinics or facilities for fall-related injuries. Also, findings from this study suggest programs for falls should especially focus on women and rural residents. Furthermore, there are only two National Centres of Aging (NCAs) in New Delhi and Chennai which provide fall-related services to adults aged ≥ 75 years; however, it would be pertinent and timely to establish more such facilities in other parts of the country. Future studies are warranted to explore this association further.

### Strengths and limitations

We used a representative sample of older Indian population from nationally representative data which permitted us to make a country-representative analysis providing comprehensive information on falls. To our knowledge, this is the first study that contributes to understanding the association of falls with multimorbidity. However, this study is limited by its cross-sectional design which cannot be used to derive the causality. Secondly, assessment of chronic conditions and falls were self -reported which may undermine the actual prevalence.

## Conclusion

We observed a considerable prevalence of falls among the older adults. Multimorbid individuals are at significant risk of experiencing falls. These findings call for prioritizing falls as a public health problem with a need to integrate falls into existing geriatric healthcare programs and future policy. Additionally, HWCs should be strengthened to incorporate fall-related services.

## Supplementary Information

**Additional file 1:** **Supplementary Figure S1. **Selection criteria for study population. **Supplementary Table S2. **Morbidity profile among study participants. **Supplementary Table S3. **Associationbetween falls and selected individual chronic conditions

## Data Availability

The dataset analysed during the current study is available in the LASI data repository held at ICT, IIPS [https://g2aging.org/?section=overviews&study=lasi].

## Abbreviations

LASI: Longitudinal Ageing Study India
LMICs: Low-and-middle Income Countries
DALY: Disability-Adjusted Life Years
HICs: High-Income Countries
MPCE: Monthly per Capita Expenditure
AOR: Adjusted Odds Ratio
NCAs: National Centres of Ageing
HWCs: Health and Wellness Centres
NCDs: Non Communicable Diseases
