# Supplementary material for: Is multimorbidity associated with higher risk of falls among older adults in India?
Source: BMC Geriatr. 2022 Jun 4;22:486. doi: 10.1186/s12877-022-03158-5 (PMC9167508; doi:10.1186/s12877-022-03158-5)
Supplement: Supplementary file 1 — Additional file 1: Supplementary Figure S1. Selection criteria for study population. Supplementary Table S2. Morbidity profile among study participants. Supplementary Table S3. Associationbetween falls and selected individual chronic conditions [file 12877_2022_3158_MOESM1_ESM.docx]

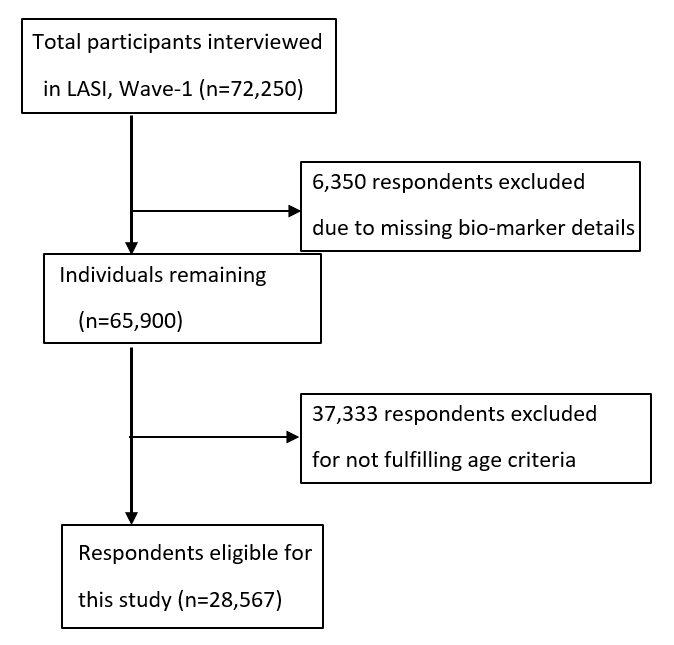


**Supplementary Figure S1: Selection criteria for study population**

**Supplementary table S2: Morbidity profile among study participants.**

| **Chronic conditions** | **n, %** | **(95% CI)** |
| --- | --- | --- |
| **Hypertension**  (n= 28,567) | 15934, 55.8 | (55.2-56.4) |
| **Neurological conditions**  (n=28,567) | 750, 2.6 | (2.4-2.8) |
| **Hearing impairment**  (n=28,567) | 2377, 8.3 | (8.0-8.6) |
| **Bone or joint diseases**  (n=28,567) | 5602, 19.6 | (19.2-20.1) |
| **Visual impairment**  (n=28,567) | 1892, 6.6 | (6.3-6.9) |
| **Obesity**  (n=28,050) | 6212, 22.2 | (21.7-22.6) |
| **Chronic heart disease**  (n=28,567) | 1507, 5.3 | (5.0-5.5) |
| **Stroke**  (n=28,567) | 720, 2.5 | (2.3-2.7) |
| **Diabetes**  (n=28567) | 4097, 14.3 | (13.9-14.8) |
| **Chronic lung disease**  (n=28,567) | 2382, 8.34 | (8.0-8.7) |
| **Cholesterol**  (n=28,567) | 723, 2.5 | (2.4-2.7) |
| **Cancer**  (n =28,567) | 197, 0.7 | (0.6-0.8) |
| **Skin diseases**  (n =28,567) | 1515, 5.3 | (5.0-5.6) |
| **Gastrointestinal problems**  (n =28,567) | 5474, 19.2 | (18.7-19.6) |
| **Chronic renal disease**  (n =28,567 ) | 1325, 4.6 | (4.4-4.9) |
| **Thyroid disease**  (n=28,567) | 635, 2.2 | (2.1-2.4) |
| **Multimorbidity (**n=28,567) | 14610, 51.1 | (50.6-51.7) |

**Supplementary table S3: Association between falls and selected individual chronic conditions**

| **Chronic conditions** | **Categories** | **Falls**  **AOR ( 95% CI)** |
| --- | --- | --- |
| **Hypertension** | Yes | 1.04 (0.92-1.18) |
|  | No | Reference |
| **Neurological Conditions** | Yes | 1.07 (0.75-1.52) |
|  | No | Reference |
| **Hearing impairment** | Yes | 1.14 (0.94-1.37) |
|  | No | Reference |
| **Bone/joint disorders** | Yes | 1.18 (1.02-1.37) |
|  | No | Reference |
| **Visual impairment** | Yes | 1.13 (0.92-139) |
|  | No | Reference |
| **Obesity** | Yes | 0.86 (0.74-1.01) |
|  | No | Reference |
| **Chronic heart disease** | Yes | 0.85 (0.64-1.13) |
|  | No | Reference |
| **Stroke** | Yes | 1.45 (1.02-2.06) |
|  | No | Reference |
| **Diabetes** | Yes | 1.09 (0.92-1.30) |
|  | No | Reference |
| **Chronic lung disease** | Yes | 0.93 (0.75-1.15) |
|  | No | Reference |
| **Cholesterol** | Yes | 1.52 (1.09-2.12) |
|  | No | Reference |
| **Cancer** | Yes | 1.22 (0.60-2.46) |
|  | No | Reference |
| **Skin diseases** | Yes | 1.64 (1.28-2.09) |
|  | No | Reference |
| **Gastrointestinal problems** | Yes | 1.50 (1.32-1.72) |
|  | No | Reference |
| **Chronic renal disease** | Yes | 1.50 (1.20-1.87) |
|  | No | Reference |
| **Thyroid disease** | Yes | 0.90 (0.60-1.35) |
|  | No | Reference |

*adjusted for age, sex, residence, wealth index, alcohol and tobacco consumption
